# Immune Responses after Vascular Photodynamic Therapy with Redaporfin

**DOI:** 10.3390/jcm9010104

**Published:** 2019-12-31

**Authors:** Ana Catarina S. Lobo, Lígia C. Gomes-da-Silva, Paulo Rodrigues-Santos, António Cabrita, Manuel Santos-Rosa, Luís G. Arnaut

**Affiliations:** 1 CQC, Chemistry Department, University of Coimbra, 3004-535 Coimbra, Portugal; catarinalobo13@gmail.com; 2 Immunology Institute, Faculty of Medicine, University of Coimbra, 3004-504 Coimbra, Portugal; paulo.santos@fmed.uc.pt (P.R.-S.); msrosa@fmed.uc.pt (M.S.-R.); 3 Laboratory of Immunology and Oncology, Center for Neuroscience and Cell Biology (CNC), University of Coimbra, 3004-504 Coimbra, Portugal; 4 Center of Investigation in Environment, Genetics and Oncobiology (CIMAGO), Faculty of Medicine, University of Coimbra, 3004-504 Coimbra, Portugal; 5 Coimbra Institute for Clinical and Biomedical Research (iCBR), Faculty of Medicine, University of Coimbra, 3004-504 Coimbra, Portugal; 6 Center for Innovation in Biomedicine and Biotechnology (CIBB), University of Coimbra, 3004-504 Coimbra, Portugal; 7 Anatomic Pathology Department, Faculty of Medicine, University of Coimbra, 3004-504 Coimbra, Portugal; amscabrita@gmail.com

**Keywords:** photodynamic therapy, redaporfin, anti-tumour immunity, neutrophilia, CD8^+^ T cells

## Abstract

Photodynamic therapy (PDT) relies on the administration of a photosensitizer (PS) that is activated, after a certain drug-to-light interval (DLI), by the irradiation of the target tumour with light of a specific wavelength absorbed by the PS. Typically, low light doses are insufficient to eradicate solid tumours and high fluence rates have been described as poorly immunogenic. However, previous work with mice bearing CT26 tumours demonstrated that vascular PDT with redaporfin, using a low light dose delivered at a high fluence rate, not only destroys the primary tumour but also reduces the formation of metastasis, thus suggesting anti-tumour immunity. This work characterizes immune responses triggered by redaporfin-PDT in mice bearing CT26 tumours. Our results demonstrate that vascular-PDT leads to a strong neutrophilia (2–24 h), systemic increase of IL-6 (24 h), increased percentage of CD4^+^ and CD8^+^ T cells producing IFN-γ or CD69^+^ (2–24 h) and increased CD4^+^/CD8^+^ T cell ratio (2–24 h). At the tumour bed, T cell tumour infiltration disappeared after PDT but reappeared with a much higher incidence one day later. In addition, it is shown that the therapeutic effect of redaporfin-PDT is highly dependent on neutrophils and CD8^+^ T cells but not on CD4^+^ T cells.

## 1. Introduction

Photodynamic therapy (PDT) combines a photosensitizer (PS) molecule, red or infrared light and molecular oxygen, none of them being individually toxic, to treat solid tumours with selectivity and reduced adverse effects. The PS is administered, after a drug-to-light interval (DLI) the target tissue is illuminated with light absorbed by the PS, and it reacts with molecular oxygen to generate reactive oxygen species (ROS). The oxidative stress locally generated by PDT is highly cytotoxic to cancer cells and to other stromal cells, such as the endothelial cells from angiogenic blood vessels [1,2]. However, PDT preserves the collagen matrix and is associated with a good cosmetic and functional outcome.

The mechanism of PDT depends considerably on the nature of the PS and on the DLI between the PS administration and the illumination. A DLI ≥ 24 h allows for the distribution of the PS to various tissues at the time of irradiation, and the tumour cells are predominantly killed by the photocytotoxicity of the PS inside the cells (cellular-PDT). The irradiation of the target tissue at DLI < 30 min finds the PS in the vasculature (vascular-PDT) and leads to the occlusion of the tumour vasculature with the subsequent tumour hypoxia, starvation and death [3]. At intermediate DLI, the PS may be also substantially retained within endothelial cells, and approaches that take advantage of this localization can be called endothelial-PDT [4]. We recently explored the combined impacts of the polarity of bacteriochlorin photosensitizers and of DLIs on the outcome of PDT [4]. More hydrophilic bacteriochlorins showed shorter plasma lifetimes and lower cellular uptake, and are more appropriate for use at shorter DLIs. More lipophilic bacteriochlorins can have longer plasma lifetimes and higher accumulation in cells, and are indicated for longer DLIs. Amphiphilic bacteriochlorins seemed to be adequate for both vascular- and cellular-PDT, and naturally also for endothelial-PDT.

PDT is not just a local treatment of solid tumours by photocytotoxicity and/or occlusion of the tumour vasculature. PDT also stimulates the host immune system, which contributes to the obliteration of any surviving cancer cells at the irradiated tumour [5,6,7,8,9,10,11] and to the recognition and destruction of cancer cells at distant locations [12,13,14]. PDT of basal cell carcinoma provided uncontroversial clinical evidence for the enhancement of systemic anti-tumour immunity [15]. The impact of PDT on the host immune system depends on the PDT regimen (e.g., DLI, light fluence, light fluence rate) and may range from immune enhancing [8] to immunosuppressive [16]. Typically, low light doses and low fluence rates are described to be more prone to stimulate anti-tumour immunity [8,17,18].

Redaporfin (i.e., 5,10,15,20-tetrakis (2,6-difluoro-3-*N*-methylsulfamoylphenyl) bacteriochlorin [19]) is an amphiphilic bacteriochlorin in clinical trials for head and neck cancer [20]. Vascular-PDT with redaporfin (0.75 mg/kg, DLI = 15 min, 50 J/cm^2^ @ 130 mW/cm^2^) enabled 86% cures of BALB/c mice with subcutaneously (sc) CT26.WT implanted tumours but no cures were achieved in immunocompromised nu/nu mice [14]. Although the redaporfin-PDT protocol applied low light doses (50 J/cm^2^) and high fluence rates (130 mW/cm^2^), 67% of the cured mice were protected from developing a new tumour after re-challenge with the same cancer cells on the contra-lateral thigh. Additionally, a significant reduction of distant lung metastasis was noted [14]. Other insightful investigations on the stimulation of anti-tumour immunity with vascular-PDT include the studies by Hamblin and co-workers using verteporfin [10,21,22] and by Scherz and co-workers using padeliporfin [5]. These studies showed that a functional immune system is essential for successful vascular-PDT, and that vascular-PDT stimulates T-cell dependent anti-tumour immunity.

Recently, it was also demonstrated that redaporfin has tropism for the endoplasmic reticulum (ER) and the Golgi complex triggering signs of ER stress and the main hallmarks of immunogenic cell death namely: the translocation of calreticulin to the cell surface, active release of ATP, the exodus of HMGB1 and the phosphorylation of eIF2α. In accordance, PDT-killed cancer cells injected subcutaneously into syngeneic mice were able to protect a fraction of the animals against a second re-challenge with live cancer cells of the same type [23,24]. Currently, redaporfin is in phase I/II clinical trials for head and neck cancer and recently, it was described the case of a patient with advanced head and neck squamous cell cancer, non-responsive to surgery, radiotherapy or chemotherapy, that had strongly benefited from redaporfin-PDT. Three sessions of redaporfin-PDT were enough to destroy all the visible tumour which in further combination with an immune checkpoint blocker (Nivolumab, PD-1) allowed a complete response with the patient, two years later after the PDT, exhibiting no signs of the disease [20].

Photodynamic stimulation of the immune system may drive PDT to the frontline of cancer immunotherapy [25]. The critical role of the immune system to the outcome of redaporfin-PDT motivated the design of this study, which aimed at investigating the mechanism of immune stimulation triggered by redaporfin-PDT, with a special emphasis on vascular-PDT. The CT26.WT implanted tumour model was selected for this investigation in view of the evidences of immune system stimulation mentioned above [14].

Numerous studies in cancer immunotherapy (including within the PDT field) have described anti-tumour immune responses. Most of the studies focused mainly on the immune responses at the tumour microenvironment and spleen lymphocytes. However, it was recently demonstrated that effective tumour eradication requires a systemic immune response, which is critical for the therapeutic outcomes [26]. The detection of systemic immune responses may also offer better opportunities for clinical translation. Monitoring anti-tumour immunity after PDT in a clinical setting is facilitated by the accessibility of blood sampling. In view of the importance of systemic immune responses and possible availability of blood samples, our study aimed at detecting signs of immune modulation at the blood of mice submitted to a vascular protocol of redaporfin-PDT. The changes in populations of neutrophils, CD8^+^ T cells and CD4^+^ T cells observed in the peripheral blood further motivated an assessment of the depletion of such cells 

## 2. Experimental Section

### 2.1. Cell Line

CT26.WT cells (ATCC CRL-2638) were cultured in Dulbecco’s Modified Eagle’s medium (DMEM) (Sigma-Aldrich, Saint-Louis, MO, USA) supplemented with 10% (*v*/*v*) heat-inactivated foetal bovine serum (GIBCO™, Life Technologies, Bleiswijk, The Netherlands), 100 U/mL penicillin, and 100 ng/mL streptomycin (Invitrogen™, Thermo Fisher Scientific, Grand Island, NY, USA). 

### 2.2. Mouse Tumour Model and PDT

The Portuguese Animal Health Authority approved the animal experiments (DGAV authorization 0420/000/000/2011). Tumours were established by sc injection of 350,000 CT26.WT cells in the right flank of BALB/c (Charles River Laboratories, Barcelona, Spain) mice ca. 10 weeks old (20 g). The optimization of vascular-PDT (0.75 mg/kg, DLI = 15 min, 50 J/cm^2^ @ 130 mW/cm^2^, 13 mm diameter illumination circle) with redaporfin was described elsewhere [14]. The illumination employed an Omicron laser at 748 nm (Omicron-Laserage, Rodgau-Dudenhofen, Germany). At different time points after tumour irradiation, mice were anesthetized and the blood was collected by abdominal artery puncture into heparin-containing tubes. 

### 2.3. Lymphocyte Analysis by Flow Cytometry 

Leukocytes (20 µL of blood) were stained with the following antibodies: APC anti-CD45, BV 605 anti-GR1, PE anti-F4/80, BV 785 anti-CD11b, Alexa Fluor 647 anti-CD3ε, PE/Cy7 anti-CD4, Alexa Fluor 488 anti-CD8a, PE/Cy5.5 anti-CD19, APC/Cy7 anti-CD11c, Pacific blue anti-CD49b, PerCP/Cy5.5 anti-CD69 and APC/Cy7 anti-CD25. Erythrocytes were depleted with BD FACS Lysing solution (BD Biosciences, San Jose, CA, USA) and cells were washed 3× with phosphate buffer saline (PBS). All antibodies were purchased from Biolegend (San Diego, CA, USA). For each sample, 20,000 lymphocytes were acquired using a FACS Canto II flow cytometer (BD Biosciences) or a Novocyte 3000 flow cytometer (ACEA, San Diego, CA, USA).

### 2.4. Quantification of Blood Cytokines 

Plasma was isolated (2000 rpm; 10 min) from the blood collected at different time points after tumour irradiation. Quantification of IL-2, IL-4, IL-6, IFN-γ, TNF, IL-17A and IL-10 was performed using the BD Cytometric Bead Array (CBA) mouse Th1/Th2/Th17 cytokine kit (BD Bioscience, San Jose, CA, USA) following the manufacturer’s instructions. 

### 2.5. Analysis of Blood Lymphocytes Expressing TNF-α, IFN-γ, IL-4 or IL-17A by Flow Cytometry

PBMC were stained at their surface as previously mentioned followed by intracellular staining with specific antibodies against TNF-α, IFN-γ, IL-4 or IL-17A cytokines. The IL-17A and IL-4 antibodies were conjugated to PE, whereas TNF-α and IFN-γ antibodies were conjugated to PerCP/Cy5.5. FIX & PERM^®^ kit (Invitrogen ™, Thermo Fisher Scientific, Grand Island, NY, USA) was used for cell fixation and permeabilization. For each sample, 20,000 lymphocytes were acquired and further analysed as described above.

### 2.6. In Vivo Depletion of Neutrophils and CD4^+^ or CD8^+^ T Lymphocytes 

Neutrophils depletion was attained with ip administrations of anti-mouse Ly6G/Ly6C monoclonal antibodies (clone NIMP-R14, BioXcell, West Lebanon, NH, USA) that were performed one day before PDT (200 µg/animal) and repeated immediately after and 5 days after irradiation (100 µg/animal). A control group with administrations of IgG isotype (clone LTF-2, BioXcell, West Lebanon, NH, USA) was also included. Blood samples were collected by tail vein puncture 24h after the first administration and neutropenia was confirmed by flow cytometry. Neutralization of CD4^+^ and CD8^+^ populations were achieved with regular ip administration of 500 µg/animal of anti-mouse CD4 (GK1.5, BioXcell, West Lebanon, NH, USA) and CD8 (53-6.7, BioXcell, West Lebanon, NH, USA) monoclonal antibodies, respectively. Each antibody was administered 24 h before the PDT protocol and its administration was repeated every five days until the end of the assay. Depletion of the target cells was confirmed by flow cytometry in blood samples 24 h after the administration of each antibody. PDT treatments were performed as abovementioned. Tumours were measured twice a week with a caliper and the volume was calculated using the formula V = (a × b^2^)/2, where a corresponds to the major diameter and *b* to the minor diameter. 

### 2.7. Histology and Immunohistochemistry (IHC)

Tumours were fixed in formalin (10%) and then embedded in paraffin. Sections of 4 μm were stained with hematoxylin and eosin (H and E) for histological analysis. Image J software was used in the blind evaluation of the necrotic areas present in the tumour sections. The evaluation is expressed as the percentage of the necrotic area in the field of view of each section. For IHC, paraffin slices of tumours were deparaffinized and hydrated. Antigen retrieval was done in 0.1 M citrate buffer (Dako Products, Agilent, Santa Clara, CA, USA). Endogenous peroxidase was blocked with 10 min incubation with 3% H_2_O_2_. Samples were then blocked with 10% goat (for anti-CD3) or rabbit (for anti-Pax5) serum and incubated, overnight at 4 °C, with a CD3 or Pax5 antibody (Dako Products, Agilent, Santa Clara, CA, USA). After washing, for CD3 staining, sections were incubated with anti-rabbit EnVision+ System-HRP Labelled Polymer (Dako Products, Agilent, Santa Clara, CA, USA) whereas for Pax5 staining, sections were incubated with a biotinylated secondary antibody, washed and incubated again with HRP containing avidin-biotin complex (VECTASTAIN ABC kit, Vector Laboratories, Peterborough, UK). All sections were revealed with 3,3’-diaminobenzidine and counterstained with Harris’ haematoxylin. Two blinded observers recorded both the total number of cells and the number of CD3^+^ cells in two sections of each tumour separated by at least 600 µm.

### 2.8. Statistical Analysis

The results are presented as the mean ± standard deviation (SD). One-way ANOVA with Dunnett’s post-test was used to determine statistically significant differences of the means between the control group and the treated groups. Survival analysis was performed by means of a Kaplan–Meier estimator (GraphPad Prism 8.0.2 Software, San Diego, CA, USA). Statistical differences were presented at probability levels of *p* < 0.05 *, *p* < 0.01 ** and *p* < 0.001 ***.

## 3. Results

### 3.1. Redaporfin-PDT Induces Accentuated Neutrophilia and Increased Levels of the Pro-Inflammatory Cytokine IL-6

**Figure 1:**
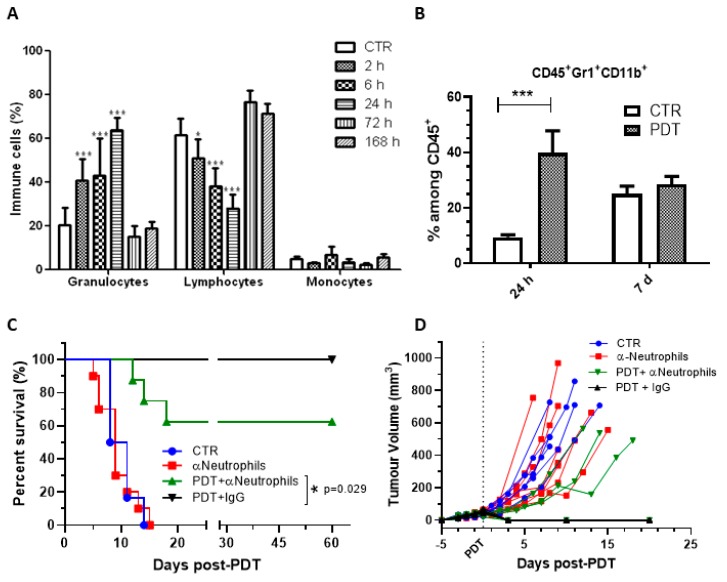
Redaporfin-PDT induces a strong neutrophilia, which contributes significantly for the treatment efficacy. (**A**) Relative percentage of blood leukocyte evaluated by flow cytometry at different time points after redaporfin-PDT. (**B**) Relative percentage of neutrophils (CD45^+^, GR1^+^ and CD11b^+^) evaluated by flow cytometry 24 h and seven days after redaporfin-PDT. Bars are the mean ± SD of six mice. No symbol *p* > 0.05; * *p* < 0.05; ** *p* < 0.01; *** *p* < 0.001. (**C**) Survival curve of mice bearing CT26.WT tumours treated with redaporfin-PDT in normal conditions or upon neutrophils depletion using Ly6G/Ly6C monoclonal antibodies. (**D**) Tumour growth represented individually for each mouse (6–11 mice per group). Survival curve statistics by LogRank (Mantel-Cox) test. No symbol: *p* > 0.05; * *p* < 0.05.

Redaporfin-vascular-PDT is currently in phase I/II clinical trials for head and neck cancer which prompted the use of Balb/c mice bearing CT26.WT (head and neck) tumours as the preclinical model. Mice were treated with redaporfin-vascular-PDT (0.75 mg/kg, DLI = 15 min, 50 J/cm^2^, 130 mW/cm^2^, 13 mm diameter illumination circle) has previously described [14]. At the indicated time points after tumour irradiation, blood samples were collected and different immune cell populations and cytokines were quantified. Our results demonstrated that redaporfin-PDT induced a sustained and significant rise in the frequency of granulocytes on the peripheral blood, which peaked 24 h post-PDT (64 ± 6%) and recovered to pre-treatment values 72 h after the treatments (15 ± 5%) (Figure 1A). Further evaluations using specific antibodies (GR1^+^ and CD11b^+^) allowed identifying that the major change in the number of granulocytes were due to a 4.2-fold increase in the percentage of neutrophils within the CD45^+^ (common lymphocyte marker) population (Figure 1B). The importance of neutrophilia for vascular-PDT with redaporfin was further assessed by depleting this population through the ip administration of monoclonal antibodies against Ly6G/Ly6C one day before PDT and twice post-PDT (immediately after irradiation and 5 days later). Flow cytometry analysis of blood samples confirmed an effective depletion of Gr1^+^ neutrophils (Figure S1), which was correlated with a significant decrease (37.5%) of the mice survival upon PDT treatments (Figure 1C,D). These results are in agreement with other studies using different photosensitizers [27,28,29]. 

**Figure 2:**
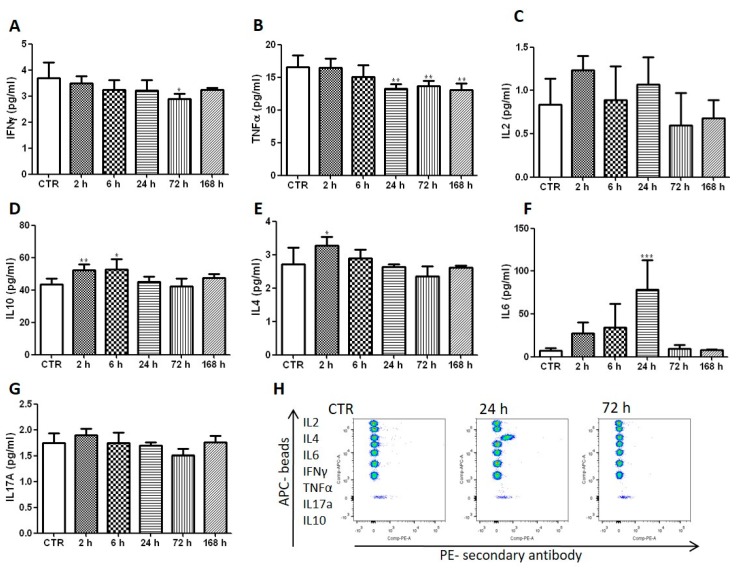
Redaporfin-PDT increases the blood levels of the pro-inflammatory cytokine IL6. The quantification of different cytokines (**A**) IFN-γ; (**B**) TNF-α; (**C**) IL-2; (**D**) IL-10; (**E**) IL-4; (**F**) IL-6; (**G**) IL-17A) was performed in the blood at different time points after vascular-PDT. (**H**) Representative dot plots depict the different cytokines in untreated and treated mice. Bars are the mean ± SD of five mice. No symbol: *p* > 0.05; * *p* < 0.05; ** *p* < 0.01; *** *p* < 0.001.

Vascular-redaporfin-PDT inflicts damage to the primary tumour, which is rapidly followed by a strong inflammation within the first hours that is known to be important for the activation of anti-tumour immunity [3]. This inflammation is expected to alter the normal leukocyte production at the bone marrow by favouring granulopoiesis over lymphopoiesis [30] which, indeed, supports the pronounced neutrophilia observed 2–24 h post-PDT. In accordance with the oedema observed in vivo upon PDT, a strong increase of the pro-inflammatory IL-6 cytokine was founded. Its levels were 11-fold higher at 24 h after vascular-PDT than those found in untreated mice (Figure 2). Although increased IL-6 levels are often reported 4–6 h after PDT [9,31], the high fluence rate used in this study (130 mW/cm^2^) is typically associated with low IL-6 expression [18]. The changes in IL-6 levels obtained with redaporfin-vascular-PDT, together with those observed for 2-[1-hexyloxyethyl]-2-devinyl pyropheophorbide-a (HPPH)-PDT using DLI = 24 h and 112 mW/cm^2^ [18], reveal that IL-6 production occurs more prominently for short DLI, which may contribute to overcome the “non-inflammatory” effect of high fluence rates. Importantly, elevated serum IL-6 is also observed in patients treated with PDT [32,33,34] and has been correlated with a better prognostic in patients (with primary bile duct cancer) submitted to treatment with hematoporphyrin-PDT [32].

The anti-inflammatory IL-10 cytokine changed in a similar manner, but its increase was rather modest (Figure 2). IL-10 prevents DC maturation and activation cytotoxic T cells [35] but, as will be shown below, the small and short-lived IL-10 increase was insufficient to prevent the production of IFN-γ by DC or by CD4^+^ and CD8^+^ T cells. In fact, increased IL-10 levels may reflect a compensatory anti-inflammatory response to limit dangerous over-reactive immune responses, thus reducing collateral tissue damage [36]. 

### 3.2. Redaporfin-PDT Activates the Adaptive Immune System and Depends on CD8^+^ T Cells for Tumour Eradication

The successful transition from innate (non-specific) to adaptive (antigen-specific) immunity determines the therapeutic outcome of different PDT regimens. This prompted us to evaluate the peripheral CD4^+^ and CD8^+^ T cells, which are highly specialized cells of the adaptive immune system. 

**Figure 3:**
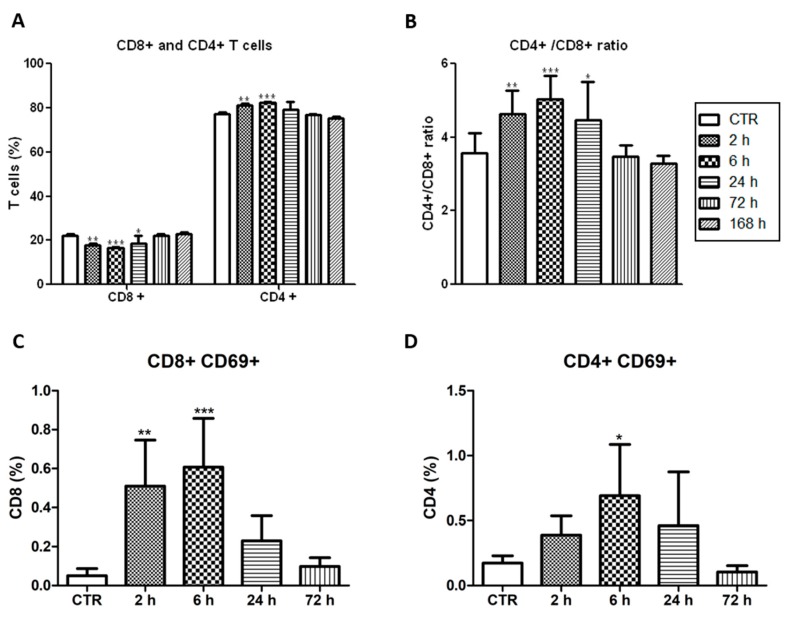
Activated T cells after vascular-PDT with redaporfin. (**A**) Percentage of CD4^+^ and CD8^+^ T cells and (**B**) ratio of CD4^+^/CD8^+^ T cells in the blood of mice at different time points after vascular-PDT. (**C**) Percentage of CD8^+^ or (**D**) CD4^+^ T cells expressing CD69 in the blood of mice at different time points after PDT. Bars are the mean ± SD of five mice. No symbol: *p* > 0.05; * *p* < 0.05; ** *p* < 0.01; *** *p* < 0.001.

CD4+ T cells (also known as helper T cells) recognize the tumour-associated antigens on the surface of antigen-presenting cells (e.g., dendritic cells, DC) and release cytokines that have a role on the regulation of the adaptive immunity. CD8^+^ T cells (also known as cytotoxic T cells) recognize specific antigens (e.g., tumour-associated antigen) in cells, which turn on their ability to induce death of those cells (e.g., cancer cell) [25]. The vascular protocol herein described presented higher CD4^+^/CD8^+^ T cells ratio within the first hours after tumour irradiation (Figure 3A,B). Importantly, higher CD4^+^/CD8^+^ T cells ratios have been correlated with increased survival rate in cancer patients [37]. The Very Early Activation Antigen, CD69, which regulates the early events of T cell activation, was upregulated both on CD4^+^ and CD8^+^ T cells. It peaked 6 h after vascular-PDT with 12-fold and 4-fold increases of CD8^+^CD69^+^ and CD4^+^CD69^+^ T cells, respectively (Figure 3C,D). This means that the ratio of the activated CD4^+^CD69^+^ and CD8^+^CD69^+^ T cells is significantly reduced 6 h post-PDT.

**Figure 4:**
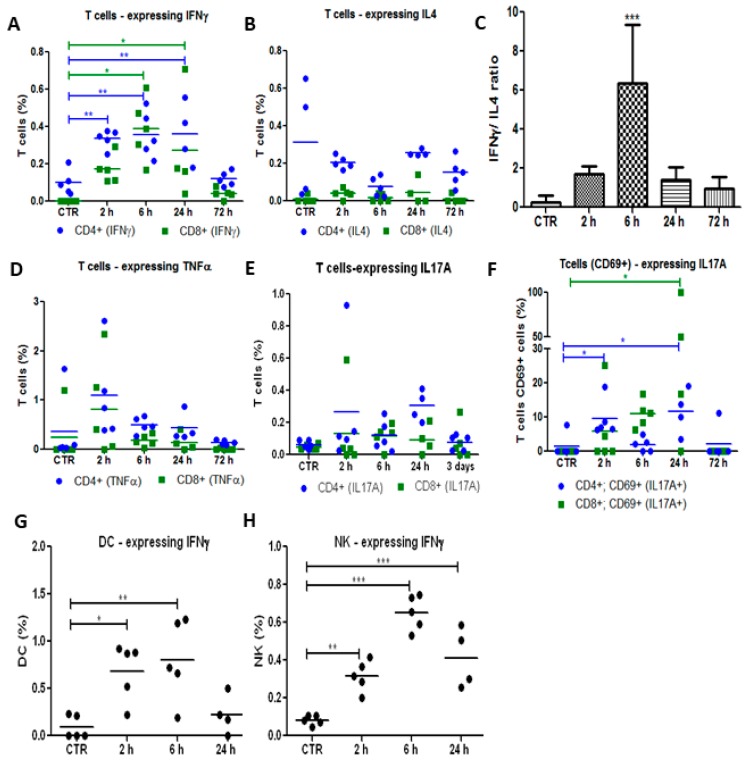
Redaporfin-PDT stimulates the production of IFN-γ and IL-17A by different immune cells. Production by T cells CD4^+^ (•) or CD8^+^ (▪) of (**A**) IFN-γ, (**B**) IL-4, (**D**) TNF-α and (**E**)**,** (**F**) IL-17A at different time points after redaporfin-PDT. (**C**) IFN-γ/IL-4 ratio, obtained by dividing IFN-γ-secreting CD4^+^ T cells by the IL-4-producing CD4^+^ T cells. (**G**) IFN-γ production by DC and (**H**) by NK. Bars are the mean ± SD of five mice. No symbol: *p* > 0.05; * *p* < 0.05; ** *p* < 0.01; *** *p* < 0.001.

Then, the production of different cytokines (IFN-γ, IL-4, TNF-α and IL-17-A) by peripheral immune cells was evaluated at different time points after tumour irradiation. Adaptive immunity can be classified in Th1 and Th2 responses. IFN-γ is the most important marker of Th1 cells, which are important for the elimination of cancer cells and virus- or bacteria-infected cells. IL-4 is secreted by Th2 cells and is typically associated with the differentiation of B cells and antibody production. Our results demonstrated that the population of CD4^+^ and CD8^+^ T cells secreting IFN-γ increased significantly in the first 24 h after vascular-PDT as well as the ratio between IFN-γ-secreting CD4^+^ T cells and IL-4-producing CD4^+^ T cells (Figure 4A–C). These findings strongly suggest the involvement of the Th1 arm of the adaptive immune system and the activation of CD8^+^ cytotoxic T cells after redaporfin-vascular-PDT. The Th1 cytokine IFN-γ has the ability to stimulate phagocytic activity of macrophages and DCs, and to coordinate the transition from innate immunity to adaptive immunity [38]. A significant increase in the percentage of IFN-γ-expressing DC and NK cells was also observed 6 h after vascular-PDT (Figure 4B), which is consistent with the ability of DC, NK and NKT cells of the innate immune system to produce IFN-γ [39]. T helper 17 (Th17) cells are a subset of T cells with the ability to produce the pro-inflammatory IL-17 cytokine, which has an important role in the migration of neutrophils into the inflammation site and in the stimulation of the granulopoiesis at the bone marrow [40], as well as in the recruitment of IFN-γ-producing CD8^+^ T cells by the tumour [41]. In fact, our results suggest that IL-17A-producing T cells slightly increased after PDT. This effect was more pronounced in the activated CD69^+^ subset of T cells (Figure 4E,F). Altogether, Th1 CD4^+^ T cells, CD8^+^ cytotoxic T cells, NK and DC cells, along with their characteristic production of IFN-γ, are important anti-tumour effectors of our vascular-PDT protocol with redaporfin. 

**Figure 5:**
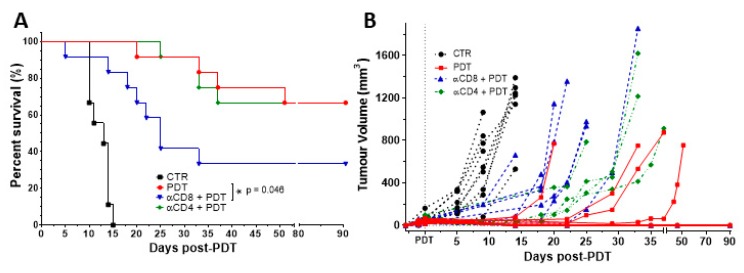
Tumour eradication by redaporfin-PDT is dependent on CD8^+^ T cells but not on CD4^+^ T cells. (**A**) Survival curve of mice bearing CT26.WT tumours treated with redaporfin-PDT in normal conditions or upon depletion of CD4^+^ or CD8^+^ T cells. (**B**) Tumour growth represented individually for each mouse (9–12 mice per group). Survival curve statistics by LogRank (Mantel–Cox) test. No symbol: *p* > 0.05; * *p* < 0.05.

The importance of CD4^+^ and CD8^+^ populations for treatment efficacy was then evaluated by depleting these cell populations through the i.p. administration of specific antibodies against CD4 or CD8. Each antibody was administered 24 h before the PDT protocol and its administration was repeated every five days until the end of the assay. Flow cytometry analysis of blood samples confirmed an effective depletion of the target cells (Figure S2). A significant impact was observed when CD8^+^ population was depleted (*p* = 0.046), with a reduction in the cure rate by half. In contrast, the depletion of CD4^+^ population had a minimal impact on the tumour growth kinetics that was not statistically significant (Figure 5). These findings suggest that cytotoxic T cells have a major role in the development of the anti-tumour immune response elicited by redaporfin-PDT, while helper T cells may have just a supportive role. It is interesting to recall that there is a higher increase of activated CD8^+^CD69^+^ T cells in the blood after PDT than of CD4^+^CD69^+^ T cells.

### 3.3. Redaporfin-PDT Changes T Cells Population in the Tumour Bed but Not B Cells

**Figure 6:**
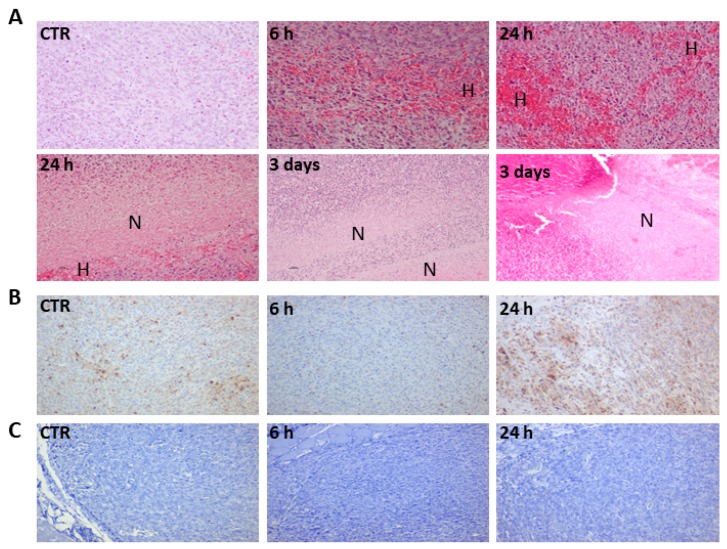
Redaporfin-PDT induces strong haemorrhage and necrosis that is accompanied by T cells infiltration but not by B cells infiltration (10x magnification). (**A**) Tumours from control and treated mice (at the indicated time points) were stained with H and E, H indicates haemorrhagic areas and N indicates necrotic areas. (**B**) T cells (CD3^+^) (brown) infiltration. (**C**) Absence of B cell (Pax5) infiltration.

Immunohistochemistry of tumours subject to vascular-PDT showed strong haemorrhage and necrosis within 24 h after vascular-PDT, which is consistent with a treatment regime that targets the tumour vasculature (Figure 6A). This observation is in agreement with our previous work that demonstrated the formation of a necrotic eschar that covers the illuminated area 3–4 days after vascular-PDT [3]. Tumour necrosis is evident 24 h post-PDT. T cells infiltration almost disappeared 6 h post-vascular-PDT, however, it re-appeared 24 h post-vascular-PDT and reached a higher level than in untreated tumours (Figure 6B) [14]. These observations are clear but qualitative, because the quantification of CD3^+^ cells in the tumour was not attempted. CD3^+^ cells were found mostly in the tumour rim, although some CD3^+^ cells were also found inside the tumour. In contrast, B cell tumour infiltration was not observed (Figure 6C). 

## 4. Discussion

The therapeutic dose found in phase I/II clinical trials for head and neck cancer with redaporfin-vascular-PDT (0.75 mg/kg, DLI = 15 min, 50 J/cm^2^ @ 130 mW/cm^2^) [20] is the same as the optimal dose found in preclinical studies with BALB/c mice bearing CT26.WT tumours (0.75 mg/kg, DLI = 15 min, 50 J/cm^2^, 130 mW/cm^2^, 13 mm diameter illumination circle), that cured 86% of the animals and led to the majority of cured animals rejecting re-challenge with the same tumour model [14]. This motivated further use of this animal-model to study immune responses after redaporfin-PDT. Although the use of more than one cell line is desirable to draw general conclusions on anti-tumour immunity, the appropriate guidance for clinical translation of redaporfin-PDT previously given by immunogenic CT26.WT tumours under the same laser fluence and dosing regimens, may also apply to immune responses in the same clinical setting. Anti-tumour immunity after redaporfin-PDT was shown using other cell lines, such as TC1 lung cancer cells [23]. 

The successful transition from innate to adaptive immunity depends on the PDT regimen and determines its efficacy. Vascular-redaporfin-PDT inflicts damage to the primary tumour, which is rapidly followed by an acute inflammation. This inflammation alters the normal leukocyte production at the bone marrow by favouring granulopoiesis over lymphopoiesis [30], a response that supports the pronounced neutrophilia observed 2 to 24 h post-PDT. This neutrophilia significantly contributes for the efficacy of vascular-redaporfin-PDT as the cure rate decreased from 100% to 62.5% when the neutrophils were systemically depleted. These results are in agreement with other studies that show neutrophilia after PDT, namely with Photofrin and (tetra(m-tetrahydroxyphenyl)chlorin (mTHPC) [27,28,29]. Selective depletion of neutrophils was previously demonstrated to reduce significantly the cure rate of Photofrin-based PDT (DLI = 24 h) [27]. This is explained by the importance of neutrophils for the stimulation of anti-tumour CD8^+^ T-cell responses, as demonstrated in one study using the photosensitizer 2-[1-hexyloxyethyl]-2-devinyl pyropheophorbide-a [42].

Consistent with the oedema/inflammation observed in vivo upon PDT, a strong increase of the pro-inflammatory IL-6 cytokine was found in the blood of mice treated by redaporfin-PDT. This result was unanticipated as typically PDT treatments at high fluence rates (as the ones used with our redaporfin-PDT regime, 130 mW/cm^2^) are associated with low IL-6 levels and with minimal anti-tumour effects. IL-6 is often found upregulated in cancer [43] and has been associated with the tumourigenic process [44]. However, IL-6 may also have an important anti-tumour role, for instance, by coordinating the transition from neutrophil to lymphocytes infiltration at the tumour bed, thus, leading to the resolution of inflammation and the initiation of T cell-mediated anti-tumour immunity [43,45]. Indeed, studies with porfimer sodium or with HPPH-PDT demonstrated that IL-6 inhibition significantly impairs the therapeutic outcome of the PDT treatment [9,29]. Additionally, increased levels of serum IL-6 have been observed in patients with oesophageal squamous cell carcinoma seven days after PDT with porfimer sodium (DLI = 48 h) [46], in patients with bile duct cancer submitted to hematoporphyrin-PDT [32] and in patients with head and neck squamous cell carcinoma 12 h after PDT with Foscan (DLI = 96 h) [33], emphasising the clinical relevance of IL-6 levels. 

We also found at the peripheral blood that redaporfin-vascular-PDT elicits an immune response mediated by CD4^+^ and CD8^+^ T cells producing IFN-γ. The Th1 cytokine IFN-γ has the ability to stimulate phagocytic activity of macrophages and DCs, and to coordinate the transition from innate immunity to adaptive immunity [38]. Other studies, both with cellular-PDT [6,22,42] and vascular-PDT [5,10,22,47] have reported T cell differentiation and enhanced IFN-γ levels after PDT. Redaporfin-PDT efficacy was dependent on T cells CD8^+^ but not on CD4^+^. Similar observations have been reported for PDT with Photofrin [11,13], which was shown to be depend on NK cells, and not on CD4^+^ cells, for the activation of T cells CD8^+^ cells. It is tempting to speculate that the same occurs for redaporfin-PDT yet it remains to be investigated in further detail. The caveat in this study is that mice cured in immune cell depletion groups were not re-challenged with the same tumour cell line to confirm, in particular, the dependence of tumour growth control on effective anti-tumour memory CD8^+^ T cells. Interestingly, Ossendorp and co-workers found that depletion of CD4^+^ T cells in a PDT protocol with DLI = 6 h (“endothelial-PDT”) led to an improved treatment outcome [48,49], whereas in our vascular-PDT the depletion of CD4^+^ T cells did not have an impact in the treatment.

Our results, combined with what is known on the enhancement of anti-tumour immunity by PDT, support the following hypothesis. Neutrophilia (Figure 1) and the strong increase of the pro-inflammatory IL-6 cytokine (Figure 2), which are related to innate immunity, are a non-specific response that occurs within the first hours after tissue damage (acute and sterile inflammation) or pathogen infection. It is well known that neutrophils are the first innate immune responders to PDT, and are followed by the recruitment of tumour infiltrating DCs. These act as mediators between the innate immune system and the adaptive immune system. Their main role is to process antigens from the tumour cells and present them on their cell surfaces to lymphocytes initiating adaptive immunity. This process seems to be accelerated in the context of redaporfin-PDT by the induction of immunogenic cell death. Redaporfin-PDT causes the rapid release of cell death-associated molecules that trigger innate immune activation and bridge toward adaptive immunity. In fact, redaporfin-PDT promotes ATP secretion, translocates calreticulin from the endoplasmic reticulum to the cell surface and releases HMGB1 more rapidly than traditional chemotherapy [23,24]. The release/exposure of DAMPs (calreticulin, HMGB1, ATP, IFN) by cancer cells dying after PDT stimulates the presentation of tumour antigens by dendritic cells and polarizes T cell response towards the production of IFN-γ, which are essential for anti-tumour immune responses [50,51,52]. DCs migrate to lymph nodes where they prime tumor-specific cytotoxic CD8^+^ T cells (adaptive immunity). Activated CD8^+^CD69^+^ T cells (Figure 3) can establish immunological memory and may kill cancer cells outside the illumination field. Depletion of CD8^+^ T cells has a dramatic effect on the efficacy of redaporfin-PDT (Figure 5).

Overall, our work demonstrates that redaporfin-vascular PDT induces extensive tissue damage at the primary (irradiated) tumour, which triggers an acute local inflammation characterized by IL-6 expression and neutrophilia that attained a maximum 24 h post-PDT. T cells expressing CD69 attained their maximum at 6 h post-PDT and IFN-γ^+^ cells were significantly over-expressed up to 24 h post-PDT, which altogether demonstrates a rapid stimulation of the immune system. B cells were not detected 2 h post-PDT, which may influence the CD4^+^ T cell proliferation [53]. At the same time, the CD3^+^ T cells are depleted at the tumour bed but later, at 24 h post-PDT, a notorious new infiltration of CD3^+^ T cells is attained. The therapeutic effect of redaporfin-PDT is dependent on neutrophils and CD8^+^ T cells but not on CD4^+^ T cells. Redaporfin-PDT is able to stimulate CD8^+^ T cells even in the absence of CD4^+^ T cells, similarly to Photofrin-PDT [13]. The dilemma between tumour-controlling (optimally curative but minimally inflammatory and ineffective to inhibit secondary disease) and immune-enhancing (inflammatory but unable to control primary tumour growth) PDT regimens [8,18] may be solved with redaporfin-vascular-PDT at high fluence rates (51 ± 2 J/cm^2^ at 130 mW/cm^2^). The effect of currently available immunotherapies seems to be limited by the absence of T cell-based inflammation [54]. Arguably, major benefits might be achieved with immunostimulating approaches that induce appropriate tissue-based inflammation. Redaporfin-vascular-PDT in a pro-inflammatory regimen achieved a successful transition from innate to adaptive anti-tumour immunity and transformed the immunosuppressive tumour microenvironment into a more favourable homing for anti-tumour immunity. This therapy may offer new opportunities to improve systemic cancer treatments.

## Supplementary Materials

The Supplementary Materials are available online at https://www.mdpi.com/2077-0383/9/1/104/s1.
