# Supplementary material for: Immune Responses after Vascular Photodynamic Therapy with Redaporfin
Source: J Clin Med. 2019 Dec 31;9(1):104. doi: 10.3390/jcm9010104 (PMC7027008; doi:10.3390/jcm9010104)
Supplement: Supplementary file 1 [file jcm-09-00104-s001.pdf]

Supplementary Materials: The following are available online at www.mdpi.com/xxx/s1, Figure S1. In vivo neutrophil depletion was confirmed by flow cytometry, Figure S2. In vivo CD4+ and CD8+ T cell neutralization was confirmed by flow cytometry.

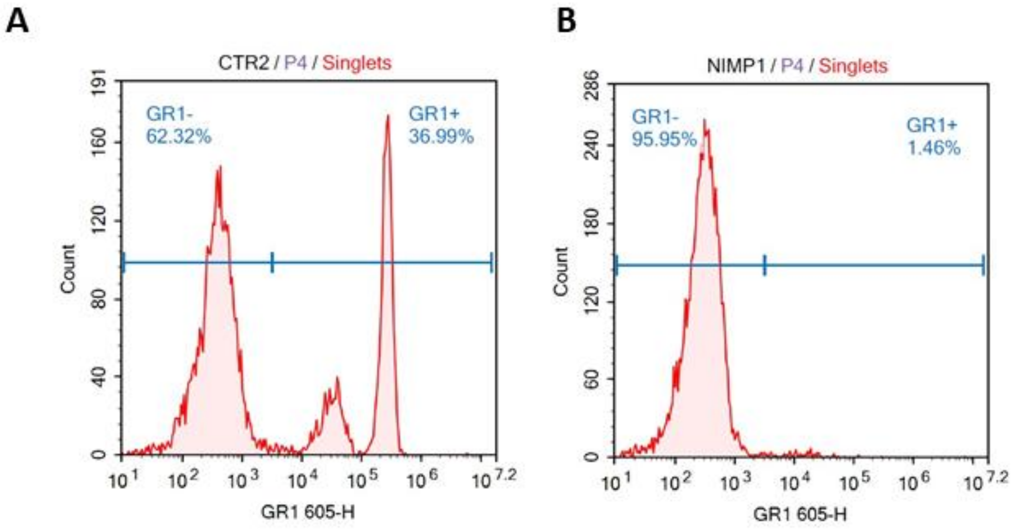

Figure S1. *In vivo* neutrophil depletion was confirmed by flow cytometry. Neutrophils depletion was attained with ip administrations of anti-mouse Ly6G/Ly6C monoclonal antibodies (NIMP-R14, BioXcell). Blood samples were collected by tail vein puncture 24 h after the first administration and neutropenia was confirmed by flow cytometry. (**A,B**) Representative histograms that show the neutrophils level ($Gr1^+$ cells) of a control and a neutrophil depleted animal, respectively.

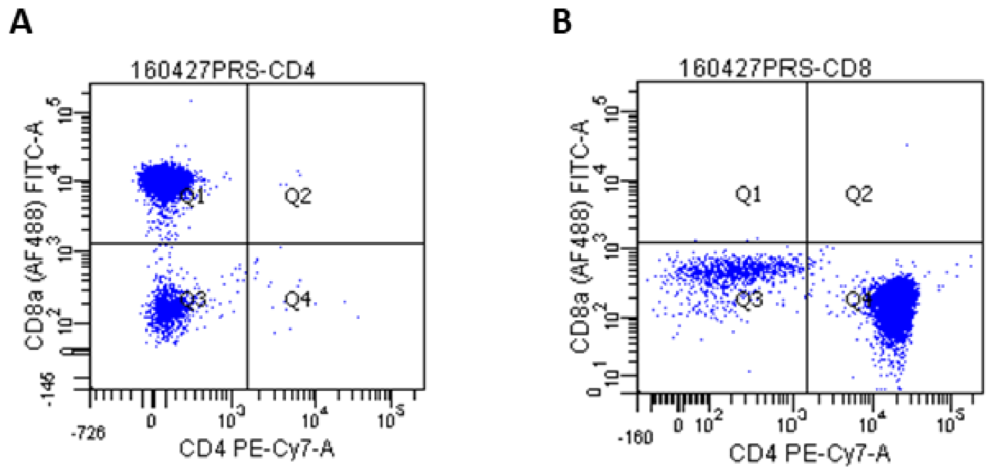

Figure S2. *In vivo* $CD4^+$ and $CD8^+$ T cell neutralization was confirmed by flow cytometry. Neutralization of $CD4^+$, $CD8^+$ were achieved with regular ip administration of anti-mouse CD4 (GK1.5, BioXcell) and CD8 (53-6.7, BioXcell) monoclonal antibodies, respectively. Specific depletion was confirmed by flow cytometry of blood samples collected 24 h after the first administration. (**A,B**) Representative dot plots that show the effective depletion of $CD4^+$ T and $CD8^+$ T cells populations, respectively.
